# The male handicap: male-biased mortality explains skewed sex ratios in brown trout embryos

**DOI:** 10.1098/rsbl.2016.0693

**Published:** 2016-12

**Authors:** P. Morán, L. Labbé, C. Garcia de Leaniz

**Affiliations:** 1 Universidad de Vigo, Vigo 36310, Spain; 2 IFREMER-INRA, BP 117, 29450 Sizun, France; 3 BioSciences, CSAR, Swansea University, Swansea SA2 8PP, UK

**Keywords:** embryo development, sex-specific mortality, sex ratio, genetic incompatibility, genetic conflict, sex determination

## Abstract

Juvenile sex ratios are often assumed to be equal for many species with genetic sex determination, but this has rarely been tested in fish embryos due to their small size and absence of sex-specific markers. We artificially crossed three populations of brown trout and used a recently developed genetic marker for sexing the offspring of both pure and hybrid crosses. Sex ratios (SR = proportion of males) varied widely one month after hatching ranging from 0.15 to 0.90 (mean = 0.39 ± 0.03). Families with high survival tended to produce balanced or male-biased sex ratios, but SR was significantly female-biased when survival was low, suggesting that males sustain higher mortality during development. No difference in SR was found between pure and hybrid families, but the existence of sire × dam interactions suggests that genetic incompatibility may play a role in determining sex ratios. Our findings have implications for animal breeding and conservation because skewed sex ratios will tend to reduce effective population size and bias selection estimates.

## 1. Introduction

The extent to which parents can control the sex of their offspring has long been the subject of much debate [1]. Fisher’s principle of equal sex allocation [2] posits that sex ratios should be roughly equal at birth because any large deviation from 1 : 1 will be quickly selected against, i.e. producing the same number of sons and daughters is an evolutionary stable strategy [3]. However, this assumes that sex allocation has no costs, which may not be the case [4]. For example, in species where maternal effects determine embryo survival and males vary more in reproductive success than females (as is typical of salmonids and many other fish [5]), mothers in good condition may be expected to produce an excess of sons, whereas mothers in poor condition should produce an excess of daughters [6]. Yet, theories of sex allocation have rarely been tested in highly fecund fishes, due to the difficulty of sexing small embryos and the absence of sex-specific markers. The recent description of the master sex-determining gene sdY in rainbow trout [7] has made it possible for the first time to sex salmonid fishes at an early stage [8]. This presents an unprecedented opportunity in evolutionary ecology because skewed sex ratios are typical of many exploited fish populations [9], and these may vary widely from year to year [10]. Testing predictions of optimal sex allocation is typically confounded by sex differences in life-history traits [11]. For example, females are generally more common among migratory fish [12] than among resident fish, which tend to be sexually balanced [13] or be male-biased [14].

At the molecular level, there is evidence that both intragenomic and intergenomic ‘genetic conflict’ can affect sex ratios, particularly when recombination patterns differ widely between the sexes [15]. Recombination rates in salmonids tend to be higher in females than in males—and perhaps most importantly—tend to occur in different chromosome regions, crossovers between homeologous chromosomes having being observed only in the telomeric regions of males [16]. Consequently, the Y-chromosome tends to accumulate more deleterious mutations, which may have consequences for embryo survival. This would present opportunities for any effects of genetic incompatibility to differ between the sexes.

We artificially crossed three hatchery populations of brown trout, a species with genome duplication and an unusually high number of chromosomes (2*n* = 80; NF = 100 [17]) in order to examine the influence of outcrossing and parental identity on sex ratios. Our expectation was that hybrid and pure crosses might differ in sex ratios due to differences in pairing and recombination between homeologous chromosomes, and that males might suffer increased embryo mortality due to their lower recombination rates and greater interference at telomeric regions.

## 2. Material and methods

We employed a partial factorial mating design [18] to cross 15 males with 15 females from three domesticated brown trout populations (F = Hardy, S = Hardy-Prosper, G = Gournay) on 5 December 2014, so that one clutch from each female was crossed with three males (one from each population). This was repeated five times to produce 15 pure and 30 hybrid families (electronic supplementary material, table S1), which were distributed in duplicate over 95 egg boxes along three tanks (mean egg density/box = 117 ± 1.6 s.e.) at the PEIMA hatchery (France). The three populations have been maintained under culture since 1986, and differ with respect to domestication selection (S, selected for growth; F and G, unselected) and genetic structure (F and S, single origin populations; G, multiple origins population [19]). Embryo mortalities were removed daily, and alevins were reared at 11.4–12.1°C for 51 days until the ‘swim up’ stage (i.e. before the onset of external feeding), at which point they were humanely euthanized (Ethical approval B2977702, 07/2013) and stored in ethanol for genetic analysis. Sex was determined using primers SdYE1S1 and SS sdYE2AS4 [7], identification being resolved by the presence of PCR product around 600–700 bp in males and its absence in females. To verify the accuracy of this method, 30 male parents from each stock, and all the female parents, were genetically sexed, and one male which failed the PCR amplification was discarded and not used in the crosses. For quality control, approximately 200 PCRs were randomly repeated and 30 embryos were sexed twice in a double blind fashion.

We employed generalized linear mixed modelling (GLMM) to analyse sex ratios using the *glmer* function in the *lme4* package of R 3.3.2. We used type of cross (pure versus hybrid), relative fecundity (egg mass/body mass) and embryo mortality (no. dead embryos/total no. eggs) as fixed factors, and dam, sire, sire nested within dam, and egg box nested within tank as random factors. We sexed 20 fish per replicate for 69% of the families; samples with less than 12 fish per replicate were excluded, which reduced the sample size to 34 families distributed over 66 egg boxes. To determine the most plausible model, we employed a hierarchical approach based on AIC changes and backward selection using the *drop1* function in *lme4* based on the likelihood ratio test, LRT [20].

## 3. Results

**Figure 1.**
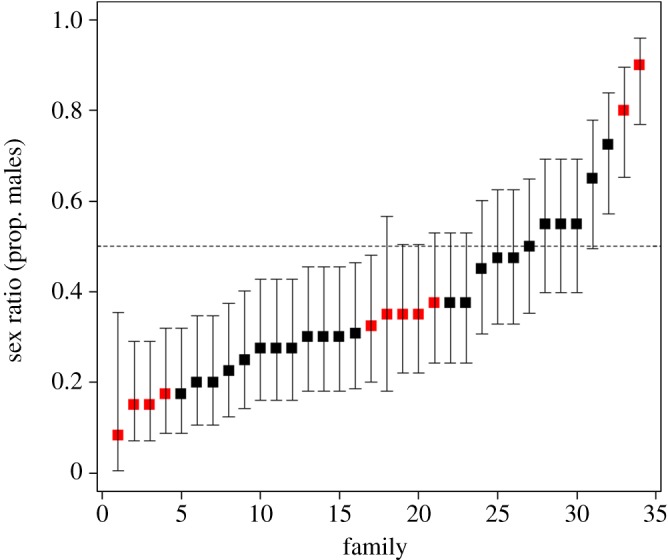
Variation in family sex ratios (proportion of males ±95 CI) for pure (red squares) and hybrid (black squares) crosses.

There was 100% agreement between phenotypic and genetic sex determination for all the parents used in the crosses, as well as for the duplicated sexing of the same embryos. We are thus confident genetic sexing was reliable. The sex ratio of 1311 embryos was significantly biased towards females (males = 501, females = 810, Fisher's Exact test *p* < 0.001). However, SR varied widely among crosses (figure 1, mean = 0.39 ± 0.03 s.e.) and 60% of families yielded significantly skewed sex ratios, these being over four times more likely to be female-biased (*n* = 17 families) than male-biased (*n* = 4 families). Family replicates (from different egg boxes) produced very similar SR (IC correlation coefficient = 0.96) and were dropped from the final model (electronic supplementary material, table S2). Hybrid crosses produced more females than pure crosses (65% versus 56%, Fisher exact test *p* < 0.001), but this was partially the result of differential mortality and could be removed from the final model (LRT = 1.813, d.f. = 1, *p* = 0.178). The most plausible SR model (table 1) included embryo mortality (estimate = −0.30, s.e. = 0.12, *p* = 0.01) and relative fecundity (estimate = −0.28, s.e. = 0.13, *p* = 0.04) as significant predictors, and sire nested within dam as a random factor (electronic supplementary material, table S2).

**Table 1.** Statistical analysis of SR (proportion of males) by GLMM obtained by backward selection. Mortality and relative fecundity were scaled prior to analysis. (Significant *p*-values are shown in bold.)

variable	d.f.	LRT	*p*-values
embryo mortality (M)	1	5.95	**0.015**
population type (T)	1	1.81	0.178
relative fecundity (F)	1	4.26	**0.039**
M × T interaction	1	2.50	0.114
M × F interaction	1	0.59	0.443
T × F interaction	1	0.15	0.701
M × T × F interaction	1	1.66	0.198

**Figure 2.**
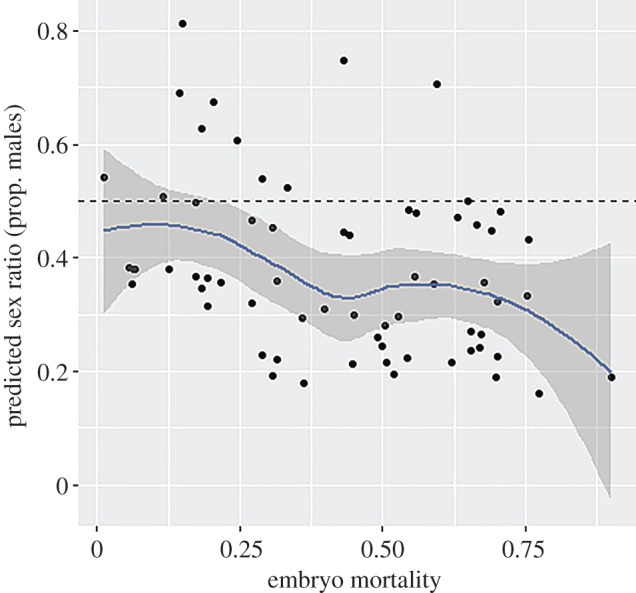
Relationship between embryo mortality and predicted SR (proportion of males) for each experimental egg box. (Online version in colour.)

Mean embryo mortality was 56.5% but this varied widely among families (range = 5–100%) and was particularly high 25–35 days post-fertilization, when 47% of mortalities occurred. Crosses with high embryo mortality were more female-biased (figure 2).

## 4. Discussion

Our study suggests that the assumption of equal sex ratios at one month post-hatching does not hold true for brown trout embryos. Instead, sex ratios were commonly skewed, these being typically female-biased, though an overrepresentation of males was also observed in some families. In common with other studies [21], our SR estimates were obtained sometime after hatching. This makes it problematic to distinguish between variation in sex allocation and sex-biased mortality. Yet, we detected significantly skewed sex ratios even in families that had sustained relatively low mortalities (13–17%), suggesting that there is scope for both. In this sense, it would be most fruitful to compare the sex ratios of live and dead embryos, though this was not possible in our study due to degraded DNA.

Skewed sex ratios appeared to be largely the consequence of high male mortality, most of which occurred during a 10-day period, corresponding to 300–450 temperature units, the stage when the teleost immune system is differentiating and salmonid embryos are most sensitive to stress [22]. This is consistent with disruption during embryo development, rather than with low fertilization success. The sex-determining locus in brown trout is located very close to the telomere of a small chromosome [23], where it may experience anomalous segregation during meiosis, result in genome imbalance [16] and impact on male survival, which is consistent with our results, though other explanations cannot be ruled out. For example, it is possible that sex ratios at birth are also skewed due to genetic conflict [15].

Contrary to our expectations, hybrid crosses did not produce offspring with more skewed sex ratios, though we found some evidence for sire × dam interactions (electronic supplementary material, table S2) and a role for maternal investment. Female-biased sex ratios were more likely among the offspring of mothers with high relative fecundity, i.e. when maternal investment was high. This suggests that parents may play a role in determining the sex ratio of their offspring, possibly through genetic incompatibility and by impacting disproportionately in the viability of male embryos, as seen in other species [24]. For example, in some birds, mating between genetically incompatible parents results in an excess of sons, thereby protecting mothers from investing in inviable daughters [25]. Likewise, some lizards sire a disproportionally high proportion of sons while others sire a large proportion of daughters [26], apparently depending on male body size.

Our findings have implications for demographic studies because survival and selection estimates that assume balanced juvenile sex ratios [27] will tend to be biased if, as our results indicate, skewed sex ratios are common early in life. Skewed sex ratios will tend to reduce effective population size [28], but the existence of sire × dam interactions means that by mating with multiple males, females may produce broods with varying sex ratios, which may represent an adaptive bet-hedging strategy [21].

## Supplementary Material

Table S1. Breeding design

## Supplementary Material

Table S2. Model selection

## Data accessibility

Data are available from the Dryad Digital Repository: http://dx.doi.org/10.5061/dryad.3h45m [29].
