# Supplementary material for: The male handicap: male-biased mortality explains skewed sex ratios in brown trout embryos
Source: Biol Lett. 2016 Dec;12(12):20160693. doi: 10.1098/rsbl.2016.0693 (PMC5206587; doi:10.1098/rsbl.2016.0693)
Supplement: Table S1. Breeding design [file rsbl20160693supp1.docx]

**Table S1.** Partial factorial mating design involving 5 sires and 5 dams per population to produce 15 pure and 30 hybrid families.

|  |  |  | **Dam** | | | | | | | | | | | | | | |
| --- | --- | --- | --- | --- | --- | --- | --- | --- | --- | --- | --- | --- | --- | --- | --- | --- | --- |
|  |  |  | **Population F** | | | | | **Population G** | | | | | **Population S** | | | | |
|  |  |  | F1 | F4 | F7 | F10 | F13 | F2 | F5 | F8 | F11 | F14 | F3 | F6 | F9 | F12 | F15 |
| **Sire** | **Population F** | M1 | X |  |  |  |  | X |  |  |  |  | X |  |  |  |  |
|  |  | M4 |  | X |  |  |  |  | X |  |  |  |  | X |  |  |  |
|  |  | M7 |  |  | 0 |  |  |  |  | X |  |  |  |  | 0 |  |  |
|  |  | M10 |  |  |  | X |  |  |  |  | X |  |  |  |  | 0 |  |
|  |  | M13 |  |  |  |  | X |  |  |  |  | X |  |  |  |  | X |
|  | **Population G** | M2 | X |  |  |  |  | X |  |  |  |  | X |  |  |  |  |
|  |  | M5 |  | X |  |  |  |  | X |  |  |  |  | 0 |  |  |  |
|  |  | M8 |  |  | 0 |  |  |  |  | X |  |  |  |  | 0 |  |  |
|  |  | M11 |  |  |  | X |  |  |  |  | X |  |  |  |  | 0 |  |
|  |  | M14 |  |  |  |  | X |  |  |  |  | X |  |  |  |  | X |
|  | **Population S** | M3 | X |  |  |  |  | X |  |  |  |  | X |  |  |  |  |
|  |  | M6 |  | X |  |  |  |  | X |  |  |  |  | 0 |  |  |  |
|  |  | M9 |  |  | 0 |  |  |  |  | X |  |  |  |  | 0 |  |  |
|  |  | M12 |  |  |  | X |  |  |  |  | X |  |  |  |  | 0 |  |
|  |  | M15 |  |  |  |  | X |  |  |  |  | X |  |  |  |  | X |

|  | pure cross |
| --- | --- |
|  | hybrid cross |

X = used in the analysis of sex ratios

0 = not used in the analysis of sex ratios due to insufficient sample size
