# Supplementary material for: The male handicap: male-biased mortality explains skewed sex ratios in brown trout embryos
Source: Biol Lett. 2016 Dec;12(12):20160693. doi: 10.1098/rsbl.2016.0693 (PMC5206587; doi:10.1098/rsbl.2016.0693)
Supplement: Table S2. Model selection [file rsbl20160693supp2.docx]

**Table S2.** Selection of (a) random and (b) fixed model structures in modelling of brown trout sex ratios.

| **(a). Random model structure** | | | | | | | | | | | | | | | | | |
| --- | --- | --- | --- | --- | --- | --- | --- | --- | --- | --- | --- | --- | --- | --- | --- | --- | --- |
| Model | | Sire | | Dam | | Sire:Dam | | | Tank:Box | | | AIC | | ∆AIC | | Comment | |
| sr1 | | X | | X | | X | | | X | | | 341.1 | | 4.0 | | failed convergence | |
| sr2 | |  | | X | | X | | | X | | | 339.1 | | 2.0 | | equivalent model | |
| sr3 | |  | | X | | X | | |  | | | 337.1 | | 0.0 | | lowest AIC | |
| sr4 | |  | | X | |  | | | X | | | 345.4 | | 8.3 | |  | |
| sr5 | |  | |  | | X | | | X | | | 339.2 | | 2.1 | | failed convergence | |
| sr6 | |  | | X | |  | | |  | | | 350.7 | | 13.6 | |  | |
| **sr7** | |  | |  | | **X** | | |  | | | **337.2** | | **0.1** | | **preferred** | |
| sr8 | |  | |  | |  | | | X | | | 354.2 | | 17.1 | |  | |
| sr9 | | X | | X | | X | | |  | | | 339.1 | | 2.0 | | equivalent model | |
|  | | | | | | | | | | | | | | |  | |  |
| **(b). Fixed model structure** | | | | | | | | | | | | | | |  | |  |
| Model | M | | T | | F | | M*T | M*F | | T*F | M*T*F | | AIC | | ∆AIC | | Comment |
| sr7 | X | | X | | X | | X | X | | X | X | | 337.2 | | 3.6 | | full model |
| sr10 | X | | X | | X | | X | X | | X |  | | 336.8 | | 3.2 | |  |
| sr11 | X | | X | | X | | X | X | |  |  | | 335.0 | | 1.4 | | equivalent model |
| sr12 | X | | X | | X | |  | X | | X |  | | 337.3 | | 3.7 | |  |
| sr13 | X | | X | | X | | X |  | | X |  | | 335.4 | | 1.8 | | equivalent model |
| sr14 | X | | X | | X | | X |  | |  |  | | 333.6 | | 0.0 | | lowest AIC |
| sr15 | X | | X | | X | |  | X | |  |  | | 335.4 | | 1.8 | | equivalent model |
| sr16 | X | | X | | X | |  |  | | X |  | | 336.3 | | 2.8 | |  |
| sr17 | X | | X | | X | |  |  | |  |  | | 334.4 | | 0.8 | | equivalent model |
| **sr18** | **X** | |  | | **X** | |  |  | |  |  | | **334.2** | | **0.6** | | **preferred** |
| sr19 | X | | X | |  | |  |  | |  |  | | 336.7 | | 3.1 | |  |
| sr20 |  | | X | | X | |  |  | |  |  | | 338.4 | | 4.8 | |  |
| sr21 | X | |  | |  | |  |  | |  |  | | 336.2 | | 2.6 | |  |
| sr22 |  | | X | |  | |  |  | |  |  | | 340.5 | | 6.9 | |  |
| sr23 |  | |  | | X | |  |  | |  |  | | 338.3 | | 4.8 | |  |
